# Spatial and temporal signatures of genomic insecticide resistance in the Anopheles arabiensis mosquito malaria vector from Ethiopia

**DOI:** 10.21203/rs.3.rs-8686648/v1

**Published:** 2026-02-11

**Authors:** Araya Eukubay, Kelly L. Bennett, Habte Tekie, Anastasia Hernandez-Koutoucheva, Fekadu Gemechu, Alistair Miles, Deriba Abera, Chris S. Clarkson, Lemu Golassa

**Affiliations:** Ethiopian Public Health Institute; Liverpool School of Tropical Medicine; Department of Zoological Sciences, Addis Ababa University; Liverpool School of Tropical Medicine; Ethiopian Public Health Institute; Ellison Institute of Technology; Aklilu Lemma Institute of Health Research, Center for Pathobiology, Addis Ababa University; Liverpool School of Tropical Medicine; Aklilu Lemma Institute of Health Research, Center for Pathobiology, Addis Ababa University

**Keywords:** Anopheles arabiensis, target site resistance, metabolic resistance, insecticide resistance, malaria mosquito vector

## Abstract

Insecticide resistance in *Anopheles* mosquitoes threatens the effectiveness of key malaria control tools such as insecticide-treated nets (ITNs) and indoor residual spraying (IRS) in Ethiopia. Genomic analysis is essential to model known and novel molecular markers of insecticide resistance for effective resistance management. This study investigated insecticide resistance genes using whole-genome sequencing in a major malaria vector, *Anopheles arabiensis* sampled across the whole regions of Ethiopia and found high geographic and temporal variability in genes associated with insecticide resistance. The Vgsc-L995F target-site substitution in the voltage-gated sodium channel gene was highly prevalent in northern Ethiopia but less common at other sites. Metabolic modes of resistance in western Ethiopia were indicated by the high frequencies of copy number variants observed at the cytochrome P450 cluster *Cyp6aa/p* and the carboxylesterase *Coeae2-7g*. Frequencies of genetic markers associated with molecular target sites and metabolic resistance were generally lower in the Central Rift Valley. However, copy number variants (CNVs) at *Gste2* and *Cyp9k1* were observed at high frequency. We observed seasonal shifts in both target-site and metabolic marker frequencies, including increasing frequencies of *Vgsc-L995F* and several cytochrome P450 variants during the major transmission season. These patterns were specific to each location. Findings indicate that molecular insecticide resistance arises from a complex interplay of factors, including malaria control interventions, agricultural practices, human behavior, and possibly vector behavior. Selection scans revealed signals of selection on chromosome 2L, centered on the *Coejhe1-5e* genes in Werkamba, northernmost Ethiopia. Additional signals were detected on chromosome 3L (~ 20 Mb), near genes that may regulate detoxification pathways, including those associated with the ubiquitin–proteasome system in Asossa, western Ethiopia. Findings highlight the importance of integrating genomic surveillance of resistance markers into entomological monitoring to strengthen insecticide resistance management. They also underscore the need to investigate lesser-known sources of adaptive change that may have significant consequences for vector control.

## Introduction

Insecticides have been crucial in preventing and controlling vector-borne diseases. However, the emergence and widespread distribution of insecticide resistance is jeopardizing their effective implementation^1^. For example, the rapid emergence of insecticide resistance in *Anopheles* mosquito vectors has contributed to the resurgence of malaria, accounting for about 19% of resurgence events according to a systematic review by Cohen et al.^2^. Within Africa, the intensity of insecticide resistance and its underlying molecular basis varies greatly between mosquito vectors, across geography and over time^3,4^. These variations are often influenced by the strength and type of insecticide applied in major malaria interventions such as insecticide-treated nets (ITNs) and indoor residual spraying (IRS)^5–7^. In addition, the distribution of insecticide resistance is also influenced by human behaviors, including personal insecticide use^8,9^, and environmental factors such as anthropogenic pollution^10,11^. Monitoring such variation in insecticide resistance is vital to the application of appropriate resistance management strategies^12,13^, but it is largely based on laboratory-intensive phenotype bioassays often coupled with assays of only a few known molecular markers^14–16^. Genomic surveillance provides a complementary or alternative approach, allowing for the rapid and efficient monitoring of known molecular markers but also the identification of novel variation under selection^17–20^. This approach is not yet routinely applied to *Anopheles* vectors in Sub-Saharan Africa^21,22^ and information on the geographical and seasonal presence of insecticide resistance is often lacking, including within the East African country of Ethiopia.

Resistance to insecticides in *Anopheles* mosquitoes primarily evolves through target-site insensitivity and changes to metabolic detoxification, although there is growing evidence that cuticular changes and behavioral adaptations also contribute^23,24^. Target-site resistance involves alterations in the genes that encode for the protein targets of insecticides. For example, amino acid substitutions in the voltage-gated sodium-channel (*Vgsc*) gene, like L995S or the linked substitutions L995F and N1570Y, confer resistance to pyrethroids and DDT^19,25^. Additionally, whole genome sequence analysis recently revealed another two linked substitutions, *Vgsc*-V402L and *Vgsc*-I1527T, which synergistically enhance *An. coluzzii* resistance to pyrethroids^17,19,26^. The primary mutations conferring resistance to dieldrin are the amino acid substitutions A296G and A296S in the gamma-aminobutyric (GABA) gene *Rdl*^27,28^. Furthermore, the substitution *Ace1*-A280S (also known as G119S) in the acetylcholinesterase-1 (*Ace-1*) gene is known to confer resistance to carbamates and organophosphates^29^, often co-occurring with gene duplications that mitigate its associated fitness cost^26,30^. Metabolic insecticide resistance mechanisms can arise from overproduction, repetition, or enhancement of detoxification enzyme genes, leading to the enzymatic breakdown or sequestering of insecticides^31^. Copy number variants (CNVs) in detoxification gene clusters, such as cytochrome P450s (*Cyp6aa/p*, *Cyp9k1*)^18^, glutathione S-transferase (*Gste2*)^32^, and carboxylesterases (*Coeae2g-6g*, *Coeae1f-2f*)^33,34^ are key drivers of resistance to pyrethroids, organophosphates, and DDT. For example, studies of *Anopheles* across West and East Africa have revealed high frequencies of *Cyp6aa/p* and *Coeae2-7g* CNVs associated with deltamethrin and pirimiphos-methyl resistance^18,33,35,36^, while duplications of the *Gste2* gene contribute to resistance to multiple classes of insecticides^26,35,37^. The wide range of insecticide resistance mechanisms present within *Anopheles* vectors highlights the dynamic nature of genomic adaptation to insecticide pressure, emphasizing that it requires regular whole-genome monitoring to inform vector control strategies.

*Anopheles arabiensis* is the primary malaria vector in Ethiopia and has a wide geographical distribution across the country^38^. This vector is found across lowland, highland, irrigated, and non-irrigated settings^39–42^, where it uses a wide range of habitats, including clear sunlit waters in permanent and temporary streams, hoofprints, artificial dams, roadside puddles, borrow pits, and rain pools^39,43–45^. The vector exhibits opportunistic, anthropozoophilic biting behavior, occurring throughout the night^46,47^. Although *An. arabiensis* shows a preference for human hosts indoors, it has a stronger inclination toward bovine hosts outdoors^47–49^. Its resting behavior is predominantly exophilic, favoring outdoor sites such as cattle sheds, pit shelters, ground holes, and vegetation over indoor resting spots. Bioassays of insecticide resistance in *An. arabiensis* have shown high resistance phenotypes to pyrethroids such as deltamethrin and permethrin, organophosphates including malathion, and the organochlorine DDT, with the levels differing across Ethiopia^14,50–53^. However, within Ethiopia, the molecular basis of insecticide resistance has been primarily investigated using targeted molecular assays of *Vgsc* knockdown down resistance mutations^14,50–53^, and gene expression studies of metabolic markers including cytochrome P450s, glutathione S-transferases, and carboxylesterases confined to a single location in the central region of the country^54,55^.

For the first time, we used whole-genome sequencing to explore the genomic landscape of the *An. arabiensis* malaria vector across the whole of Ethiopia. Moreover, we present the first comprehensive genomic analysis of this species over both the major and minor malaria transmission seasons and across multiple years. Using longitudinal data, we analysed both SNPs and CNVs to investigate the spatial pattern of known target-site and metabolic resistance markers in the *Anopheles* genome. We also applied genome-wide selection scans to identify novel markers with the potential to be under local selection pressure from insecticides.

## Methods

### Mosquito Sampling

**Figure 1:**
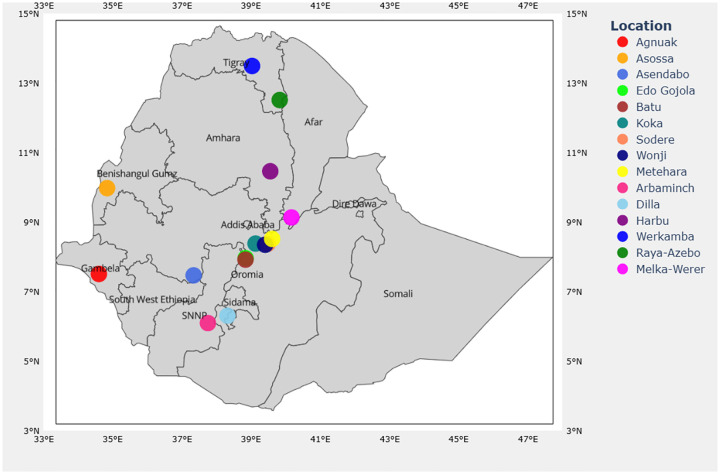
Map of Mosquito Sampling Sites across Ethiopia.

Mosquitoes were collected from fifteen sites (Fig. 1) across the northern, central Great Rift Valley, southern, southwestern, and western Ethiopia. The sites are malarious with different ecoepidemiology, where both the ITN and IRS malaria vector control have been implemented for decades. Malaria transmission in most regions of Ethiopia follows a biannual seasonal pattern, with a major peak from September to December after the main rainy season (June-September) and a minor peak from April to July following the short rainy season (March and May)^56–58^. Mosquitoes were sampled during both major (September-December) and minor (April-July) transmission seasons between 2020 and 2023. *Anopheles* mosquitoes were sampled from selected households using a CDC light trap, mouth aspiration, and prokopack aspiration, both from indoor and outdoor locations, following the standard procedures described^59^. The CDC light traps were installed from 18:00 pm to 6:00 am. Battery-assisted Prokopack and manual aspirations were of a 15-minute duration per household from 6:00 am to 8:00 am, targeting indoor walls, under furniture, and animal shelters. Larval collections were performed using a standard dipper. Larvae were reared to adults for morphological identification, and all mosquito samples were identified morphologically using the standard identification keys^60^ before individual preservation in 0.3 mL volume of PCR plates using 150 μL of 80% ethanol.

### Whole genome sequencing and processing

Mosquitoes were whole-genome sequenced as part of the Malaria Vector Genome Observatory following the *Anopheles gambiae* 1000 Genomes Project (https://malariagen.github.io/vector-data/ag3/methods.html) protocols. DNA of individual mosquito specimens was extracted using the Qiagen DNeasy Blood and Tissue kit (Qiagen Sciences, MD, USA) according to the manufacturer’s protocol. DNA was fragmented using Covaris Adaptive Focused Acoustics, and paired-end multiplex libraries were prepared as per Illumina’s instructions. All mosquito specimens were sequenced using the Illumina HiSeq X platform. Bioinformatic analysis, including raw sequence processing, quality filtering, read alignment, and variant calling, was performed using pipelines designed by the *Anopheles gambiae* 1000 Genomes project (https://malariagen.github.io/vector-data/ag3/methods.html). Reads were aligned to the AgamP4 reference genome using BWA version 0.7.15 and single nucleotide polymorphism (SNP) data generated with GATK version 3.7.0. Samples with a median coverage less than 10x, with less than 50% genome coverage, or with a high contamination threshold (> 4.5%) were excluded. CNV calling was based on copy number state generated across windows of the genome using normalized coverage data and a Gaussian Hidden Markov Model (HMM) implemented in hmmlearn (GitHub - hmmlearn/hmmlearn: Hidden Markov Models in Python, with scikit-learn like API) as previously described by Lucas et al. (2019)^18^. CNV calls were filtered such that only those with a high likelihood > 1000 predicted by the HMM model were retained. To increase CNV prediction accuracy, individuals with a high coverage variance (> 0.35) were removed. Genotypes at biallelic SNPs that met site filtering criteria were phased into haplotypes using a combination of read-backed and statistical phasing methods using WhatsHap V1.0^61^ and SHAPEIT V4.2^62^, respectively, following protocols of the *Anopheles gambiae* 1000 Genome Project https://malariagen.github.io/vector-data/ag3/methods.html).

### Insecticide Resistance

Frequencies for population cohorts organised by the location, year, and month of collection were calculated for non-synonymous amino acid substitutions using the transcript for genes associated with pyrethroid, DDT, and organophosphate resistance. These included the gene encoding for the voltage-gated sodium channel (*Vgsc*; AGAP004707), which is the target site for pyrethroids, the GABA-gated chloride channel (resistance to dieldrin) gene, which is the target site for dieldrin (*Rdl*; AGAP006028), the acetylcholinesterase gene (*Ace1*; AGAP001356), which codes for the target of organophosphates and a glutathione S-transferase gene, for which substitutions confer resistance to DDT and permethrin (*Gste2*; AGAP009194). To account for sequencing and alignment error, only substitutions at a frequency > 5% were considered. We also generated the frequencies of copy number variants (CNVs) present at greater than 5% for cytochrome P450 genes such as *Cyp6aa/p* (AGAP002862-AGAP013128)^63^ and *Cyp9k1* (AGAP000818), *Cyp9m1* (AGAP009363)^64^, *Cyp6z2* (AGAP008218)^65^, the carboxylesterases *Coeae1f/2f* (AGAP006227-AGAP006228)^34^ and *Coeae2-7g* (AGAP006723-AGAP006727)^33^, the acetylcholinesterase *Ace1* (AGAP001356)^66^, and glutathione S-transferase *Gste2* (AGAP009194)^26,30^. All the analyses were conducted in the malariagen_data Python package. A chi-square contingency test was applied to assess the statistical significance of regional patterns in either amino acid substitutions or CNV frequencies at known resistance markers using the SciPy Python package^67^. For statistical analysis, the study sites were categorized by regions according to their geographical proximity: Northern (Werkamba, Raya-Azebo, Harbu), Central (Melka-Werer, Metehara, Wonji, Koka, Batu, Edo Gojola), Southern (Dilla, Arbaminch), Southwestern (Asendabo), and Western (Agnuak, Asossa).

### Genome-wide selection scans

A genome-wide selection scan (GWSS) was performed using Garud’s H12 homozygosity statistic^68^ across windows of the genome to detect signals of recent positive selection using the malariagen_data Python package (https://malariagen.github.io/vector-data/ag3/api.html). A window size calibration was performed for each chromosome arm before H12 analysis to determine the distribution of H12 values below 0.1 for the 95th percentile. The analysis was restricted only to chromosomes two and three since the X chromosome in the *An. arabiensis* is highly divergent from the *An. gambiae* reference genome.

## Results

### Population sampling

A total of 1272 *An. gambiae* s.l. collected from fifteen sites were whole-genome sequenced at an average median sequence coverage of 34X. Specimens were identified as *An. arabiensis* based on Ancestry Informative Markers (AIM) and Principal Components Analysis (PCA) with known taxa during the standard data processing protocols of the *Anopheles gambiae* 1000G Project (https://malariagen.github.io/vector-data/ag3/api.html). A total of 69,636,442 SNPs passing site quality filters were segregated in the dataset, of which 33,534,459 were biallelic (Table 1).

### Target-site insecticide resistance variants

**Figure 2:**
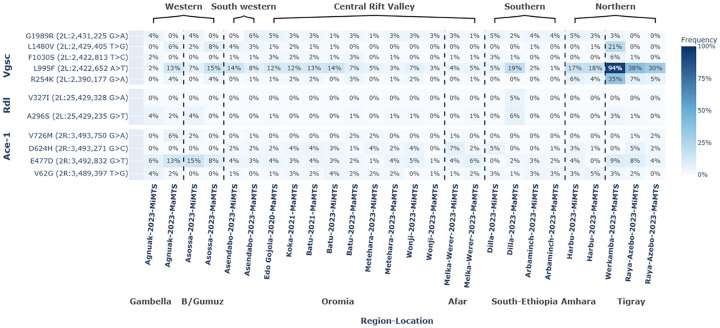
Amino acid allele frequencies for the *Vgsc* (AGAP004707-RD), *Rdl* (AGAP006028-RA) and *Ace-1* (AGAP001356-RA) genes in *Anopheles arabiensis* across different regions of Ethiopia by sampling year and malaria transmission season. The Y-axis represents the frequency of the amino acid variants, each defined by a specific amino acid change and its genomic position. **MaMTS**: major malaria transmission season; **MiMTS**: minor malaria transmission season.

To investigate the presence of target site resistance, we calculated the frequencies of non-synonymous (amino acid altering) substitutions at the voltage-gated sodium channel (*Vgsc*: gene id AGAP004707-RD), acetylcholinesterase (*Ace-1*: gene id AGAP001356-RA), and GABA-gated channel gene (*Rdl*: gene id AGAP006028-RA). The *Vgsc*-L995F associated with knockdown resistance to pyrethroids^19,69^ was detected at a high frequency in the Northern Ethiopia Tigray region, with a frequency between 38% and 94% (Fig. 2). Lower but appreciable frequencies of 13–19% were also detected in the south-central regions of Amhara and South Ethiopia, as well as the western regions of Gambella and Benshangul Gumuz. We used a chi-squared contingency test to explore whether there was a significant difference in the frequency of *Vgsc*-L995F across the different regions of Ethiopia. We found a significant difference in the *Vgsc*-L995F frequency in the northern region when compared to frequencies in the central, southern, southwestern, and western regions of Ethiopia (P < 0.05, Supplementary Table S1). All other comparisons were non-significant. In some locations, we also observed a marked difference in frequencies moving into the major transmission season, but these were location-specific. For example, in Agnuak in western Gambella and Dilla in South Ethiopia, frequencies of L955F increased by 11% and 14%, respectively. In contrast, frequencies in northern Raya-Azebo and Asendabo in the southwest decreased by 8%. Substitutions associated with insecticide resistance, including *Ace1*-G280S and *Rdl*-A296S, were not observed at > 5% frequency in all the sites except in the southern Ethiopian, Dilla town where the frequency of A296S was 6%, similar to previous observations for *An. arabiensis* from East Africa^36,70^.

### Copy number variation at known metabolic insecticide resistance genes

**Figure 3:**
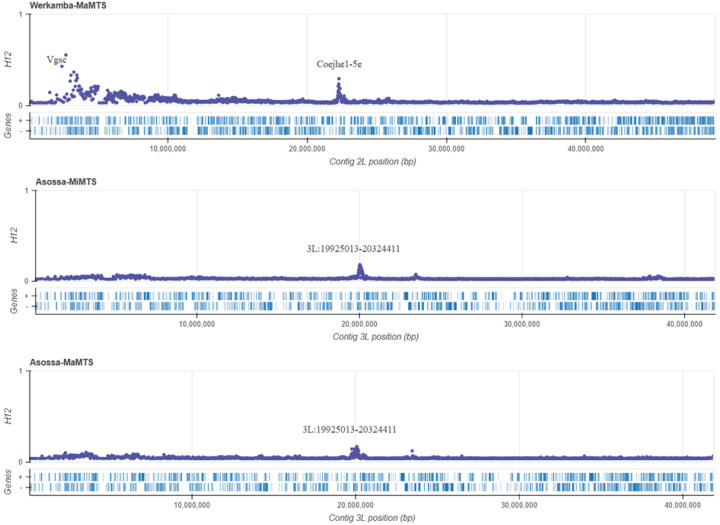
Copy number variation (CNV) frequency of the *Anopheles arabiensis Cyp450, COE*s, and *Gste2* gene clusters in *Anopheles arabiensis* from locations in Ethiopia stratified by year and malaria transmission season. The Y-axis represents the frequency of CNV amplification (amp) for each gene cluster. For the *Gste2*gene, CNV and single nucleotide polymorphisms (SNPs) are plotted together. **MaMTS**: major malaria transmission season; **MiMTS**: minor malaria transmission season.

We calculated the proportion of individuals with any number of CNV amplifications for each gene across study sites and seasons. The proportion of individuals with at least one CNV amplification at the *Cyp6aa/p* gene cluster, associated with pyrethroids and organophosphate insecticides^18,26^, was particularly high (65%) at the western site of Gambella. Furthermore, a chi-squared contingency test revealed the western region to have higher frequencies of CNVs compared to other regions in Ethiopia (P < 0.05, Supplementary Table 2, Fig. 3). In contrast, CNV frequencies at *Cyp6aa/p* were moderate or low but statistically similar (P > 0.05, Supplementary Table 2) in cohorts from Northern Ethiopia (0–29%), the Central Ethiopian Great Rift Valley (6–12%), and southwestern Ethiopia (6–7%). Moderate and statistically different frequencies were observed in Southern Ethiopia (6–29%) (P < 0.05, Supplementary Table 2). Frequencies of amplifications at the carboxylesterase *Coeae2-7g* were also comparatively high in western Gambella, with a frequency of 26% compared to less than 13% at other sites (Fig. 3). However, a chi-squared contingency test revealed that the CNV frequencies at the *Coeae2-7g* gene cluster were not significantly different for most regional comparisons, although frequencies in the western region were significantly different from that of southern Ethiopia (P < 0.05, Supplementary Table 2). CNV frequencies at other cytochrome P450s previously associated with resistance, including *Cyp9k1* and *Cyp6z2*, were generally comparable across sites (Fig. 3). *Cyp9k1* amplification was consistently high (21–65%) and, in general, statistically similar across northern, western, southern, and southwestern Ethiopia (P > 0.05, Supplementary Table 2). However, frequencies were significantly higher, reaching up to 78% in the Central Great Rift Valley (P < 0.05, Supplementary Table 2) The frequency of *Cyp6z2* CNVs remained low (0–18%) and was not statistically different across the different regions of Ethiopia (P > 0.05, Supplementary Table 2). We observed that frequencies in both *Cyp9k1* and *Cyp6z2* showed a frequency increase during the major transmission season in certain locations only, revealing that there is variation in the seasonal dynamics of metabolic resistance markers. For example, *Cyp9k1* amplifications reached 78% compared to a lower frequency of 54% during the minor transmission season in Afar (Melka-Werer). Furthermore, *Cyp6z2* CNV frequencies reached 15% in Tigray (Raya-Azebo) compared to 0% during the minor transmission season.

In contrast to observations for cytochrome P450s and carboxylesterases, frequencies of *Gste2* amplifications associated with pyrethroid and organophosphate resistance (Mitchell et al., 2014)^32^ were highest in *An. arabiensis* from the northern region (35–98%), southwestern Ethiopia (79–91%), southern Ethiopia (70–78%) and the Central Ethiopian Great Rift Valley sites (58–88%), which were statistically similar to one another for most comparisons (P > 0.05, Supplementary Table 2). Conversely, frequencies of *Gste2* were significantly lower in the western Ethiopian sites of Gambella and Benishangul-Gumuz (23–35%) (Fig. 3). Interestingly, the known SNPs of *Gste2* (L119V, I114T) associated with resistance to DDT and pyrethroids^32,71^ were absent in *An. arabiensis* from Ethiopia.

### Genome-wide selection scans

**Figure 4:**
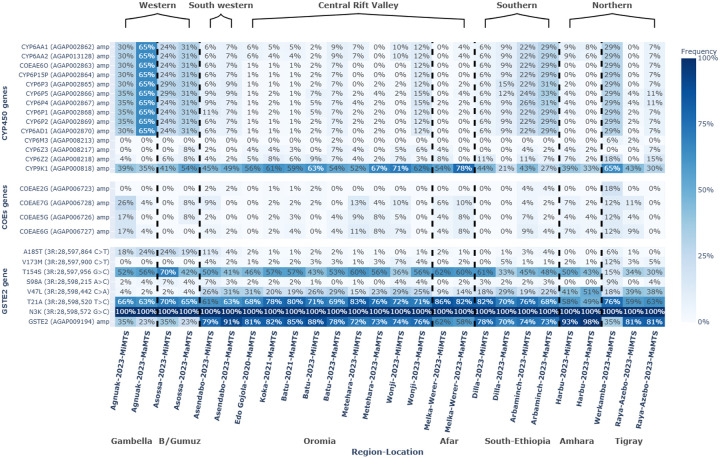
Genome-wide selection scan of the H12 homozygosity statistic across the 2L and 3L chromosomes. Peaks in H12 values are centered in the 2L on the *Coejhe1-5e*gene cluster, and 3L:19925013–20324411 region, which includes 12 protein-coding genes, indicating a putative selection of signals. The name, gene ID, and description of genes found under the sweep on 3L are provided in Supplementary Table S3. **MaMTS**: major malaria transmission season; **MiMTS**: minor malaria transmission season.

A genome-wide scan for recent selection was performed using the H12 statistic, to investigate the presence of known and novel selection signals^68^. We identified a novel signal of selection on chromosome 2L, spanning 22.27–22.33 Mb, which encompasses the genes *Coejhe1-5e*. This selection signal was restricted to the cohorts from Werkamba only (Fig. 4). We checked for CNV amplifications at these genes and found a moderate to high (33–70%) frequency at *Coejhe3e* in all cohorts but a particularly high frequency of 82% in Werkamba. Although we found a 0–17% CNV frequency at *Coejhe4e* across all cohorts, CNVs were absent in Werkamba. We did not observe CNVs at the *Coejhe1*, *2*, and *5e* genes (Supplementary Figure S1). Similarly, we identified a signal of selection close to the *Vgsc* gene in the Werkamba site only (Fig. 4), where we found a high frequency of the L995F substitution. H12 analysis also revealed a novel signal of selection centred on chromosome 3L spanning 19.9–20.3 Mb, which is approximately 400 kb. The sweep signal encompasses 12 protein-coding genes (Supplementary Table S3), including chloride channel, fibrinogen, E3 ubiquitin ligase SMURF1/2, ADP-ribosylation factor-like protein 5, and ubiquitin factor-like protein 5. Notably, these selective signatures were only found in the Asossa site in Western Ethiopia (Fig. 4), and they were absent in any of the other study locations. Similarly, we found a signal of positive selection on the 3R chromosome around 31.84 Mb, encompassing the *Cyp9m1, Cyp9m2, Gstu3*, odorant-receptor, nuclear hormone receptor *FTZ-F1 beta*, and cellular retinaldehyde-binding protein genes, in Northern Ethiopian cohorts from Raya-Azebo and Harbu and in the Wonji site located in the Central Rift Valley (Supplementary Figure S2). We screened for CNVs amplification in this region and found low frequencies at *Gstu3* ranging from 0–14% across all cohorts, including those without a selection signal. Furthermore, we did not find amplifications at the other genes (Supplementary Figure S1), suggesting that the minor peak we observed could result from other structural variants such as SNPs, insertions, or indels.

We also observed a more pronounced selection signal in the 2R chromosome centered on the *Cyp6aa/p* gene cluster (Supplementary Figure S3) in all populations of *An. arabiensis*, despite low frequencies of CNVs in cohorts from the Central Ethiopian Great Rift Valley. The clear signal of selection in the absence of a high CNV frequency suggests that an amino acid substitution (Supplementary Figure S4) or another structural variant may drive selection at this cytochrome P450. In support of the variable frequencies of *Gste2* amplifications we observed, a signal of selection was detected at the gene on 3R in *An. arabiensis* from the Central Ethiopian Great Rift Valley and northern Amhara region (Supplementary Figure S2), but not the western region. Only a weak signal was observed in the northern sites. We did not observe a difference in selection signals between the major and minor malaria transmission seasons.

## Discussion

In this study, we used whole-genome sequence analysis of 1272 *An. arabiensis* from Ethiopia to investigate molecular insecticide resistance mechanisms. We found marked geographical and temporal heterogeneity in resistance profiles across Ethiopia, pointing to potential influences of environmental and behavioral factors that warrant further investigation. Alongside established resistance markers, analysis of whole genomes allowed the identification of novel selection signals, highlighting new candidates for drivers of insecticide resistance, suggesting the need for targeted and routine monitoring to inform vector control strategies.

We found considerable geographical variation in the molecular markers of insecticide resistance across Ethiopia. The historical use of DDT-based IRS since the 1960s^72^, followed by the scale-up of pyrethroid-based LLINs since 2005^73^, has been similarly applied across all malarious areas in Ethiopia. Despite the uniform application of vector control strategies across the country, the observed geographical differences in resistance profile could be impacted by differences in agricultural pesticide runoff. For example, the high frequency of *Vgsc*-L995F and selection signal observed in northern Ethiopia is likely driven by the region’s intensive agricultural use of pyrethroids (lambda-cyhalothrin) and persistent DDT applications in horticultural crops, compounded by historical DDT exposure^74^. The western Ethiopia lowland also supports intensive large- and small-scale farming, mainly cotton and sesame^75^, and uses broad-spectrum pesticides^76–78^, which could explain the observed higher frequencies of cytochrome P450 and carboxylesterase CNVs.

We also observed a comparatively lower frequency of *Gste2* CNVs in the western region may reflect differing insecticide selection pressure. Although *Gste2* amplifications are strongly associated with DDT metabolism^71^, their current distribution is more likely shaped by fitness costs^79^, cross-resistance to pyrethroids^71,80–82^, and other ecological factors such as agricultural chemical exposure and oxidative stress environments^83,84^. Furthermore, the comparable frequency of *Cyp6aa/p* CNVs we observed in the northern, southwestern and central Great Rift Valley regions may be influenced by shared agricultural practices, as farmers in both regions cultivate vegetables and cereals and apply similar classes of pesticides^74,85^. Additionally, the implementation of comparable vector control interventions such as ITNs and IRS may contribute to uniform selection pressure. However, there are also differences in intervention coverage and the type of insecticides used at the different locations that may contribute to variation in resistance patterns. For example, in western Ethiopia, where malaria transmission is high and year-round^57^, IRS with carbamate, organophosphate, and neonicotinoid (clothianidin-based) insecticides has been implemented^41,86,87^, alternating every two years in addition to the standard LLINs intervention in selected villages. This approach may increase metabolic-based resistance frequency while reducing pyrethroid target-site resistance-conferring markers due to potential fitness costs^88,89^. Additionally, it is possible that *Anopheles* behavior contributes to differences in the frequency of insecticide resistance-conferring markers. For example, in the cooler highlands of northern Ethiopia, lower outdoor temperatures may drive higher indoor biting rates among vectors such as *An. arabiensis*^42,90^, potentially exposing *An. arabiensis* to greater selection pressure from pyrethroid-based indoor interventions such as ITNs. However, this hypothesis is not fully supported due to a lack of species-specific comparative studies determining *An. arabiensis* behavior across the sites. However, it highlights that we know little of how this behavioral trait contributes to selection for insecticide resistance. Finally, we expect geography itself to impact the presence of genomic markers of insecticide resistance. For example, although not fully supported by the statistical analysis, we observed lower frequencies of both *Vgsc*-L995F and cytochrome P450s in the Great Rift Valley compared to elsewhere, despite high frequencies of *Cyp9k1*, indicating the presence of selection for pyrethroid resistance^26,91,92^. Therefore, additionally or alternatively, it may be that restricted gene flow between the Great Rift Valley and other Ethiopian *An. arabiensis* has historically limited the spread of adaptive variation across the country. For example, genetic differentiation between the northwestern highlands and the Great Rift Valley of Ethiopia has been previously observed based on measures of F_ST_ using microsatellite markers (Nyanjom et al., 2003)^93^. The high mountains surrounding the central Rift Valley populations may restrict mosquito gene flow, as evidenced by prior studies^70,94^, underscoring the necessity for further investigation into population connectivity in *An. arabiensis*.

We observed marked differences in genomic insecticide resistance markers across the minor and major transmission seasons, demonstrating for the first time the potential of genomic data to track localised selection pressure from insecticides across a fine temporal scale. We found that seasonal differences were location-specific, and we did not have multi-year comparisons across seasons for statistical confirmation. However, we found that resistance-conferring variants (L995F and *Cyp6aa/p* CNV) increased during the major malaria transmission (September to December) in western (Gambella) and southern (Dilla, Arbaminch) cohorts. The increase was potentially driven by intense insecticide pressure from agricultural runoff^78^ and/or LLIN utilization favoring individuals carrying resistant alleles over susceptible alleles, since IRS was not applied at these locations in 2023. In contrast, in northern (Raya-Azebo) and southwestern (Asendabo) Ethiopia, resistance variants unexpectedly increased in cohorts collected in July, which is typically the onset of the major rainy season rather than during the peak transmission season (September-December). This increase is possibly driven by year-round irrigation, sustained breeding habitats, and high intervention (ITN) utilization^95^, thereby maintaining selection pressure even before the onset of major transmission. Additionally, it may be coupled with climate variability, such as erratic rainfall, that supports emerging mosquito populations exposed to residual agricultural pesticides^74,96^.

Our genome-wide selection scan revealed a novel signal of selection on the 2L chromosome in cohorts from northern Ethiopia (Werkamba). This selective sweep was centered on a gene cluster encoding five carboxylesterases: *Coejhe1e*, *Coejhe2e*, *Coejhe3e*, *Coejhe4e*, and *Coejhe5e*. While the primary function of these genes is in juvenile hormone regulation, their involvement in insecticide resistance is increasingly recognized. Indeed, there is a growing body of evidence demonstrating that members of these clusters, particularly *Coejhe2e*, are found consistently upregulated in organophosphate- and pyrethroid-resistant *An. arabiensis* populations^64,97^, suggesting their potential involvement in the metabolic insecticide resistance mechanism. We also identified a small selection peak on the 3L chromosome arm around 19.9–20.3 Mb in cohorts from Asossa in western Ethiopia. This region encompasses genes like E3 ubiquitin ligase SMURF1/2, chloride channel, ADP-ribosylation factor-like protein 5 (Arl5), ubiquitin factor-like protein 5, and fibrinogen. While these genes themselves have not yet been directly linked to insecticide resistance in *Anopheles* species, related E3 ubiquitin-protein ligase classes (such as SMURF1/2 like genes) have been identified as hub genes in resistance to organophosphate and pyrethroids in *An. arabiensis* and *An. gambiae* from Kenya and Benin^99^. Moreover, studies in yeast and *Drosophila* suggest that Arl5 and ubiquitin factor-like protein 5 support resistance by regulating stress response^100–102^. The ubiquitin factor-like protein 5 regulates mRNA splicing to assure that detoxification or antioxidant genes are expressed correctly. The Arl5 helps proteins move around the cell to keep it stable when oxidative stress is present^101,102^. Furthermore, the chloride channel ortholog, pICln-like protein, is a conserved regulator of spliceosomal snRNP assembly and cellular volume control in *Drosophila* with potential implications for gene expression under stress or developmental conditions^103^. Variants that enhance the efficiency of splicing or stress response may amplify the production of detoxification enzymes^104,105^. It is unknown whether these genes perform analogous functions in *An. arabiensis* and contribute to increased insecticide resistance, but they highlight the need for further functional investigation. In addition, we detected a small peak on the 3R chromosome centered around the *Cyp9m1, Cyp9m2*, and *Gstu3* genes previously reported to be overexpressed in pyrethroid-resistant *An. arabiensis* and *An. funestus*^64,106,107^. This peak was identified particularly in northern sites (Raya-Azebo and Harbu) and the central Great Rift Valley site of Wonji, but not in other surveyed sites. *Cyp9m1* and *Cyp9m2* encode Cyp450s commonly associated with metabolic insecticide detoxifications, while *Gstu3* belongs to the *Gste* class, implicated in conjugation-based detoxification of pyrethroid and DDT. Although these genes are functionally linked to resistance, we did not detect CNVs at the *Cyp9m1* and *Cyp9m2* loci, which are typical of cytochrome P450 metabolic resistance^26,33,34^. Although CNVs in *Gstu3* were identified at a low frequency, this was true of all study sites including those where no selection peak was observed. Our findings indicate that structural variation at this locus might be widespread but not necessarily under strong directional selection. Besides, the selection signal also comprises other genes with a potential role in insecticide resistance, including odorant receptors, the nuclear hormone receptor *FTZ-F1 beta*, and cellular retinaldehyde-binding protein^108,109^. Odorant receptors, while primarily involved in olfactory signalling, may contribute to behavioral resistance by modulating host-seeking and avoidance of insecticide-treated surfaces^108,110^. *FTZ-F1 beta, a* nuclear hormone receptor, regulates cuticular protein expression and has been shown to confer pyrethroid resistance in *Culex pipiens pallens* by modulating cuticular permeability^109^. The role of other genes in the mosquitoes is not studied. The selection signal for all aforementioned genes was minimal, but the strength of the peak could be affected by sampling effort. Further longitudinal sampling is needed to monitor the persistence of selection and confirm its potential significance in insecticide resistance. However, findings demonstrate how genomic surveillance is instrumental in identifying novel selection signals^20,22,36,70^, enabling the detection of genetic adaptations for further study that may contribute to insecticide resistance.

## Conclusion

This study presents the first whole-genome sequencing data for *An. arabiensis* from Ethiopia using trans-seasonal collections across the major and minor malaria transmission seasons and multiple years from sites representing the whole of Ethiopia. This novel effort reveals, for the first time, the importance of capturing both spatial and seasonal patterns in the genomic landscape of insecticide resistance to uncover the full extent of its variation. An understanding of fine-scale temporal dynamics is critical in offering further resolution for the tailoring of malaria vector control strategies at the right time and with targeted interventions. In particular, our findings highlight the complexity of insecticide resistance in *An. arabiensis* across Ethiopia, driven by spatially heterogeneous mechanisms hypothesized to be shaped by vector control pressures, agricultural pesticide runoff, and interactions between human and vector behavior. In the Tigray region of northern Ethiopia, a high prevalence of *Vgsc*-L995F is likely to reduce the efficacy of pyrethroid-based interventions, potentially elevating vector survival. Conversely, in the western region, elevated *Cyp6aa/p* CNV amplification and moderate L995F frequencies may undermine LLIN and IRS efficacy while possibly promoting cross-resistance to organophosphates. In the central Rift Valley, high amplification of *Cyp9k1* and *Gste2* may circumvent the efficacy of conventional ITNs and IRS despite lower *Vgsc-L995F* frequencies. Further study is required to directly link the agricultural use of pesticides and human and vector behavior to both geographical and temporal genomic resistance patterns, as well as resistance phenotypes. Such studies will be crucial to comprehensively recognise the complexity of the selection landscape and to inform evidence-based control strategies.

## Supplementary Material

1

This is a list of supplementary files associated with this preprint. Click to download.

- SupplementaryTablesFigures.docx

## Figures and Tables

**Table 1:** Summary of *Anopheles arabiensis* samples whole-genome sequences available for analysis.

Region	Sampling site	Latitude	Longitude	Year	Month	Anopheles arabiensis (N)
Gambella	Agnuak	7.504	34.293	2023	July	25
					November	27
B/Gumuz	Asossa	10.062	34.539	2023	June	23
					October	13
South Ethiopia	Arbaminch	6.033	37.583	2023	May	49
					November	49
	Dilla	6.254	38.173	2023	May	19
					October	42
Oromia	Asendabo	7.462	37.152	2023	July	50
					November	45
	Edo Gojola	7.975	38.722	2020	September	85
	Batu	7.933	38.717	2021	September	7
				2021	October	150
				2023	May	49
				2023	October	49
	Koka	8.411	39.021	2021	October	110
	Sodere	8.404	39.388	2021	September	6
	Wonji	8.376	39.311	2023	May	46
					October	6
					November	30
	Metehara	8.551	39.522	2023	June	50
					October	9
					November	40
Amhara	Harbu	10.562	39.465	2023	May	49
					November	49
Tigray	Raya-Azebo	12.667	39.75	2023	July	50
					November	28
	Werkamba	13.667	38.917	2023	November	17
Afar	Melka-Werer	9.19	40.105	2023	June	50
					October	50

## Data Availability

The sequences of the samples identified in this study were submitted to the European Nucleotide Archive (ENA; accession numbers are given in Supplementary Table S4).
