# Supplementary material for: Spatial and temporal signatures of genomic insecticide resistance in the Anopheles arabiensis mosquito malaria vector from Ethiopia
Source: Res Sq. 2026 Feb 11:rs.3.rs-8686648. Preprint. [Version 1] doi: 10.21203/rs.3.rs-8686648/v1 (PMC12919180; doi:10.21203/rs.3.rs-8686648/v1)
Supplement: 1 [file NIHPPRS8686648V1-supplement-1.pdf]

Table S1

Frequency of *Vgsc*-L995F resistance allele in *Anopheles arabiensis* from Ethiopia by geographical regions. Statistical significance was determined using a chi-square test (df = 1). A p-value ≤ 0.05 was considered significant.

| **Location** | | **χ 2** | **df** | ***p*-value** |
|---|---|---|---|---|
| Northern | Central | 129.11 | 1 | < 0.0001 |
| | Southern | 65.80 | 1 | < 0.0001 |
| | Southwestern | 27.73 | 1 | < 0.0001 |
| | Western | 33.70 | 1 | < 0.0001 |
| Central | Southern | 1.68 | 1 | 0.1956 |
| | Southwestern | 0.59 | 1 | 0.4435 |
| | Western | 0.01 | 1 | 0.9301 |
| Southern | Southwestern | 2.55 | 1 | 0.1106 |
| | Western | 0.37 | 1 | 0.5451 |
| Western | Southwestern | 0.41 | 1 | 0.5245 |

Table S2

Frequency of *Cyp6aa/p, Coeae2-7g*, *Cyp9k1*, *Gste2*, and *Cyp6z2* copy number variation (CNVs) in *Anopheles arabiensis* across geographical regions in Ethiopia. Statistical significance was determined using a chi-square test (df = 1). A p-value ≤ 0.05 was considered significant.

| **Location** | | **Genes** | | | | | | | | | |
|---|---|---|---|---|---|---|---|---|---|---|---|
| | | ***Cyp6aa/p*** | | ***Coeae2-7g*** | | ***Cyp9k1*** | | ***Gste2*** | | ***Cyp6z2*** | |
| | | **χ 2** | **p-value** | **χ 2** | **p-value** | **χ 2** | **p-value** | **χ 2** | **p-value** | **χ 2** | **p-value** |
| Northern | Central | 1.442 | 0.2298 | 0.666 | 0.4144 | 27.380 | **< 0.0001** | 1.298 | 0.2546 | 0.006 | 0.9375 |
| | Southern | 8.693 | **0.0032** | 2.551 | 0.1102 | 1.051 | 0.3052 | 4.541 | **0.0331** | 0.000 | 1.0000 |
| | Southwestern | 0.000 | 0.9914 | 0.621 | 0.4307 | 1.940 | 0.1637 | 0.000 | 1.0000 | 0.621 | 0.4307 |
| | Western | 39.773 | **< 0.0000** | 0.611 | 0.4343 | 0.012 | 0.9113 | 33.795 | **< 0.0000** | 0.023 | 0.8791 |
| Central | Southern | 32.352 | **< 0.0000** | 1.289 | 0.2562 | 36.386 | **< 0.0001** | 2.295 | 0.1298 | 0.000 | 1.0000 |
| | Southwestern | 0.202 | 0.6531 | 0.085 | 0.7707 | 4.304 | **0.0380** | 0.242 | 0.6225 | 1.169 | 0.2797 |
| | Western | 109.873 | **< 0.0000** | 3.289 | 0.0697 | 11.098 | **0.0009** | 42.437 | **< 0.0000** | 0.159 | 0.6904 |
| Southern | Southwestern | 5.909 | **< 0.0151** | 0.021 | 0.8836 | 5.104 | **0.0239** | 1.931 | 0.1646 | 0.817 | 0.3660 |
| | Western | 10.908 | **< 0.0010** | 5.302 | **0.0213** | 1.008 | 0.3153 | 12.088 | **0.0005** | 0.085 | 0.7713 |
| Western | Southwestern | 25.734 | **< 0.0000** | 2.172 | 0.1406 | 0.800 | 0.3711 | 18.550 | **< 0.0000** | 0.031 | 0.8601 |

Table S3
Putative genes showing increased H12 values (peaks) in the Asossa *Anopheles arabiensis* cohorts.

| Gene ID | Conting | Start | End | Name | Description |
|---|---|---|---|---|---|
| AGAP011227 | 3L | 19635709 | 19644016 | NaN | Tartan |
| AGAP011229 | 3L | 19810767 | 19826552 | NaN | Tartan |
| AGAP011231 | 3L | 19867709 | 19868872 | NaN | fibrinogen |
| AGAP011242 | 3L | 19935159 | 19978825 | NaN | E3 ubiquitin ligase SMURF1/2 |
| AGAP011243 | 3L | 19979246 | 19979846 | NaN | Ribonuclease H2 subunit |
| AGAP011244 | 3L | 19979876 | 19981896 | NaN | rRNA 2'-O-methyltransferase fibrillarin |
| AGAP011245 | 3L | 19982535 | 19983733 | NaN | chloride channel |
| AGAP011246 | 3L | 19984046 | 19985106 | NaN | origin recognition complex subunit 6 |
| AGAP011247 | 3L | 19986323 | 19995087 | NaN | Longitudinals lacking protein-like |
| AGAP011249 | 3L | 19999770 | 20002401 | NaN | pre-mRNA-splicing factor CWC26 |
| AGAP011251 | 3L | 20072720 | 20077159 | NaN | ubiquitin-like protein 5 |
| AGAP011254 | 3L | 20102345 | 20111903 | Arl5 | ADP-ribosylation factor-like protein 5 |

Table S4
List of sample sets and accession numbers of the sequence samples. Samples with multiple run accessions are given within their respective sample sets.

| Sample sets | Accession number |
|---|---|
| 1270-VO-MULTI-PAMGEN-VMF00162 | ERR6045394-ERR6045422,ERR6045425-ERR6045453,ERR6045456-ERR6045484 |
| 1270-VO-MULTI-PAMGEN-VMF00162 | ERR5987917-ERR5987948,ERR5987949-ERR5987980,ERR5987981-ERR5988012 |
| 1270-VO-MULTI-PAMGEN-VMF00162 | ERR5967771-ERR5967798,ERR5967799-ERR5967826,ERR5967827-ERR5967854 |
| 1270-VO-MULTI-PAMGEN-VMF00218 | ERR10419623-ERR10419646 |
| 1270-VO-MULTI-PAMGEN-VMF00218 | ERR10419672-ERR10419694 |
| 1270-VO-MULTI-PAMGEN-VMF00218 | ERR10490762-ERR10490785 |
| 1270-VO-MULTI-PAMGEN-VMF00218 | ERR10490810-ERR10490977 |
| 1270-VO-MULTI-PAMGEN-VMF00218 | ERR10970454-ERR10970494,<br>ERR11041589-ERR11041629 |
| 1324-VO-ET-GOLASSA-VMF00257 | ERR12262329-ERR12262396 |
| 1324-VO-ET-GOLASSA-VMF00257 | ERR12314213-ERR12314278 |
| 1324-VO-ET-GOLASSA-VMF00257 | ERR12325996-ERR12326278 |
| 1324-VO-ET-GOLASSA-VMF00257 | ERR12547479-ERR12547495 and ERR12767674 |
| 1324-VO-ET-GOLASSA-VMF00275 | ERR12871282-ERR12871307 |
| 1324-VO-ET-GOLASSA-VMF00275 | ERR12948031-ERR12948246 |
| 1324-VO-ET-GOLASSA-VMF00275 | ERR12983126-ERR12983194 |
| 1324-VO-ET-GOLASSA-VMF00275 | ERR13101382-ERR13101590 |
| 1324-VO-ET-GOLASSA-VMF00275 | ERR13146891-ERR13146976 |
| **Supplementary Figures** | |
